# Temporoparietal Junction Hypoactivity during Pain-Related Empathy Processing in Adolescents with Conduct Disorder

**DOI:** 10.3389/fpsyg.2016.02085

**Published:** 2017-01-11

**Authors:** Daifeng Dong, Qingsen Ming, Xiang Wang, Weixia Yu, Yali Jiang, Qiong Wu, Yidian Gao, Shuqiao Yao

**Affiliations:** Medical Psychological Institute, Second Xiangya Hospital of Central South UniversityChangsha, China

**Keywords:** conduct disorder, empathy, fMRI, temporoparietal junction, adolescents

## Abstract

**Background:** Lack of empathy has been proposed to account for the characteristic behavioral problems exhibited by adolescents with conduct disorder (CD). Hence, the aim of this study was to determine whether adolescents with CD exhibit atypical affective and cognitive neural empathic responses during pain-related empathy processing.

**Methods:** A total of 30 adolescents with a CD diagnosis and 36 without CD symptoms were recruited from out-patient clinics and local middle schools in the same region, respectively. All 66 participants were subjected to functional magnetic resonance imaging (fMRI) while viewing video clips depicting a face with a neutral expression receiving non-painful stimulation (Q-tip touch) or a face with a painful expression receiving painful stimulation (needle penetration) applied to the left or right cheek.

**Results:** The regions associated with affective and cognitive empathy were activated in the HC group during pain-related empathy processing. Compared to HCs, adolescents with CD showed significantly reduced activation in the bilateral temporoparietal junction (TPJ).

**Conclusions:** Adolescents with CD exhibited dampened hemodynamic responses during pain-related empathy processing in the bilateral TPJ, a region associated with cognitive empathy. These findings are consistent with the hypothesis that adolescents with CD may have a cognitive empathy deficiency.

## Introduction

Conduct disorder (CD) is a psychiatric disorder that emerges during childhood or adolescence. It presents repetitive and chronic aggressive and antisocial behavior in which the basic rights of others or major age appropriate norms or rules of society are violated (APA, 2000). CD has been reported to occur in about 16% of preadolescents (Olsson, 2009; Jiang et al., 2015; Zhang et al., 2015), and may co-exist with other disorders, such as attention-deficit/hyperactivity disorder (ADHD) (Rubia, 2011), oppositional-defiant disorder (Loeber et al., 2000), and substance abuse (Whitmore et al., 1997). Aggressive and antisocial behavior displayed by children with CD might reflect atypical empathic responses to others' suffering (Blair, 2005), several behavioral studies have reported findings suggesting that CD youth may be deficient in empathy (Cohen and Strayer, 1996; Blair et al., 2001; Blair, 2005; Wied et al., 2005; Schwenck et al., 2012).

Empathy is an ability to understand and resonate the affective experience of others (Preston and De Waal, 2002; Singer and Lamm, 2009; Bernhardt and Singer, 2012). According to empathy duality model (Shamay-Tsoory et al., 2009; Shamay-Tsoory, 2011, 2013; Raz et al., 2014), empathy is constructed with two separate systems, one is the affective empathy, and the other is cognitive empathy (Shamay-Tsoory, 2013). The affective empathy is defined as a bottom-up automated process associated with sharing the bodily states of others and is linked to a set of brain regions, including the inferior frontal gyrus, a cortical area that is involved with the mirror neuron system (Rizzolatti, 2005), as well as the anterior insula and the middle anterior cingulate cortex (ACC). The cognitive empathy (Shamay-Tsoory, 2013) is defined as a more top-down system that allows the making of inferences regarding the mental states of others. It has been associated with various brain regions, including the ventral and dorsal medial prefrontal cortex, the superior temporal sulcus, precuneus, and temporoparietal junction (TPJ). Although both affective empathy and cognitive empathy may operate partly autonomously, it is likely that every empathic response will evoke both kinds of empathy to some extent, depending on the social context (Shamay-Tsoory, 2011). Empathy plays an important role in inhibiting aggression and promoting prosocial behavior (Decety and Jackson, 2004; Eisenberg et al., 2005) and a lack of empathy may be an important precipitating factor of conduct problems(CP) (Decety et al., 2009). Therefore, it is important to determine whether adolescents with CD exhibit atypical empathic neural responses.

To explore the neural mechanism of empathy, about two kinds of paradigm have been developed in previous studies (Lamm et al., 2011): one is the cue-based paradigm, which used abstract visual symbols (cues) of different colors indicated whether the target person him/herself would receive electrical stimulation and whether this stimulation would be painful or not; and the other is picture-based paradigm, which used the visual displays depicting the target persons in emotional situations as experimental stimuli.

To the best of our knowledge, only few functional magnetic resonance imaging (fMRI) studies have explored the atypical neural substrate of empathy in children with CP, and their results were not consistent with each other (Decety et al., 2009; Sebastian et al., 2012; Lockwood et al., 2013; Schwenck et al., 2017). Decety et al. (2009) found that, adolescents with aggressive CD showed increased neural responses to other's pain in several regions including the insula, midcingulate cortex (a division of the ACC), striatum, temporal pole, and amygdala in relation to healthy controls (HCs). Sebastian et al. (2012) found that children with CP showed hypoactivity in amygdala and anterior insula in a complex affective processing task including an empathy component. Lockwood et al. (2013) found that children with CP showed reduced empathic responses to others' pain in bilateral anterior insula, ACC, and inferior frontal gyrus using a region of interest (ROI) analysis. More recently, Schwenck et al. (2017) found that adolescents with CP didn't show neural activation in affective empathy network while observing others winning or losing a gambling task. Despite the differences between the imaging findings of these studies, all suggested that adolescents with CD exhibited atypical empathic responses relative to HCs.

Notably, the aforementioned fMRI studies had several limitations. On the one hand, the samples recruited in the previous studies either have comorbidities with ADHD (Decety et al., 2009; Schwenck et al., 2017), or lack definite diagnoses of CD (Whitmore et al., 1997; Sebastian et al., 2012; Lockwood et al., 2013). Therefore, the conclusions of these studies may not generalize to the broader population of adolescents with CD. Besides, the sample size of previous studies were relatively small. To attain a clear view of the empathic neural responses of CD adolescents, a large sample of “pure” CD adolescents that with definite diagnoses and without comorbidities, especially ADHD, should be recruited. On the other hand, the results of previous studies (Decety et al., 2009; Sebastian et al., 2012; Lockwood et al., 2013; Schwenck et al., 2017) were inconsistent. This inconsistency might be due to the heterogeneity of the samples and distinct principal type of empathy evoked in the fMRI study.

Every empathic responses may evoke both the affective empathy and cognitive empathy to some extent, depending on the social context (Shamay-Tsoory, 2011). Generally, the paradigm of participants without being aware of the goal of experiments mainly evoked the neural responses of affective empathy (Fan et al., 2011), and the paradigm requiring explicit imagination or evaluation of feelings mainly evoked the cognitive empathy (Fan et al., 2011). According to this, most of previous related studies might mainly investigate the affective empathy. However, the social context are far complicated in the reality life, so these two kinds of empathy are usually evoked to a large extent, and several studies (Shamay-Tsoory, 2013; Raz et al., 2014) have addressed that the cognitive empathy and affective empathy have dynamic functional integration. Hence, it is meaningful to investigate the empathic neural responses in CD group with a task evoking both the affective empathy and cognitive empathy.

Based on the above descriptions, we recruited a large sample of CD adolescents without comorbidities to test whether there were differences in cognitive and affective empathic responses between adolescents with “pure” CD and HCs. For this purpose, a well-established pain-judgment empathy task (Han et al., 2008, 2009; Xu et al., 2009) with explicit evaluation of feelings was used. Electrophysiological studies (Fan and Han, 2008) have proved that the pain-judgment task could elicit both the early affective empathy and late cognitive empathy. Although fMRI studies failed to distinguish this two kinds of empathy with a pain-judgment task because of the low temporal resolution of blood oxygen dependent (BOLD) signals, the high spatial resolution of MRI provided a possibility to test whether there were atypical empathic responses in regions associated with affective empathy and cognitive empathy in CD group. Only males were recruited because of its higher prevalence rate of conduct disorders among adolescents(Loeber et al., 2000) and lower empathic ability in relative to females(Rueckert and Naybar, 2008; Derntl et al., 2010; Christov-Moore et al., 2014). We hypothesized that there would be atypical affective and cognitive empathic responses in the “pure” CD group compared to the HC group.

## Material and methods

### Samples

The CD group consisted of 30 male adolescents who were recruited from out-patient clinics affiliated with the Second Xiangya Hospital of Central South University (Changsha, Hunan, China). The HCs included 36 healthy age- and intelligence quotient (IQ)-matched male adolescents who were selected randomly from local middle schools in the same region. The Wechsler Intelligence Scale for Children (Gong and Cai, 1993) was applied to measure IQ. All 66 participants were right-handed according to the Ediburgh Handedness Inventory (Oldfield, 1971). The present study was approved by each school's administration and the Ethics Committee of the Second Xiangya Hospital of Central South University. All participants and their parents were informed of the purpose of this study and provided written informed consent to be involved in the study.

The Structured Clinical Interview for DSM-IV-TR Axis I Disorders-Patient Edition (SCID-I/P) (First et al., 2001) was administered to all participants by two well-trained psychiatrists. All participants in the CD group were confirmed to fulfill the DSM-IV-TR criteria for CD (APA, 2000). Information was collected from each patients and at least one corresponding parent to improve the reliability of the diagnostic interview. A psychiatrist made the final decision if the information provided by a patient and his parents was inconsistent. None of the HCs met the criteria for CD or any other psychiatric disorders, or had history of CD symptoms or aggression. All participants had normal or normal-corrected vision. Participants were excluded from the study if they reported any following exclusion criteria: a history of ADHD, oppositional-defiant disorder, or any psychiatric or emotional disorder; diagnosis of any pervasive developmental or chronic neurological disorder, Tourette syndrome, post-traumatic stress disorder, or obsessive compulsive disorder; persistent headaches; head trauma; a history of alcohol or substance abuse over the past year; contraindications to fMRI; or an IQ ≤ 80.

### Stimuli and procedure

The stimuli consisted of 24 video clips, each shown for 3 s, showing faces of six Chinese actors (3 males; 4 video clips of each actors). Half of the clips (2/4 of each actors) depicted a face with a neutral expression receiving non-painful stimulation (a Q-tip touch) and half (the other 2/4 of each actor) showed a face with a painful expression receiving painful stimulation (needle penetration) applied to the left or right cheek (half each side, order random). Participants were instructed to judge whether or not the actor was feeling pain by pressing a button with the right index (yes pain) or middle (no pain) finger. They could press the button in the duration of video clips or the interval of two successive video clips in each trail. Three 300-s functional scans were obtained from each subject. Each scan consisted of all 24 video clips, in this way, each video clip were shown 3 times. The inter-stimulus interval between successive clips was random (7, 8, 9, 10, and 11 s) during which participants fixated on a central cross. The 24 inter-stimulus interval of each scan included 5 interval of 7 s, 5 interval of 8 s, 4 interval of 9 s, 5 interval of 10 s, and 5 interval of 11 s. The last clip in each scan was followed by a 12-s fixation period.

After the scanning procedure, participants were shown 36 video clips again and asked to rate the pain intensity felt by the actor (“how much pain do you think the actor is feeling?”) using a Likert–type scale where 0 indicated no pain and 10 indicated maximal possible pain intensity (i.e., extremely painful). Participants performed these evaluations outside the scanner so that their empathic responses during scanning were not affected by these evaluation processes. The 36 video clips included 12 faces with a neutral expression receiving non-painful stimulation, 12 faces with a painful expression receiving painful stimulation, and 12 faces with a neutral expression receiving painful stimulation. Among them, the faces with a neutral expression receiving non-painful stimulation and faces with a painful expression receiving painful stimulation have been previously used in the fMRI task. On the one hand, the post-scanning procedure was aimed to test whether the painful and non-painful condition could be distinguished, on the other hand, this procedure was designed to assess the subjective feelings of anothers' pain.

### Self-reporting assessments

All participants completed the Interpersonal Reactivity Index Scale (IRI) (Davis, 1980, 1983), an instrument that assesses trait empathy. The strengths and difficulties questionnaire (SDQ) (Yao et al., 2009) was used to detect tendencies for internalization and externalization of problems. In addition, the Chinese version of Buss-Perry Aggression Questionnaire (AQCV) (Buss and Perry, 1992) was used as a quantitative index of aggressive tendencies. Callous-unemotional (CU) traits were evaluated with the Antisocial Process Screening Device (APSD) (Frick and Hare, 2001).

### Neuroimaging methods

#### Imaging acquisition and preprocessing

All fMRI data (repetition time/echo time = 2000/30 ms, slice thickness = 4 mm, number of slices = 32, matrix size = 64 × 64, field of view = 240 × 240 mm, flip angle = 90°) were acquired during the task using a SIEMENS Skyra 3.0-T whole body scanner at The Second Xiangya Hospital. SPM8 software (Statistical Parametric Mapping, the Welcome Trust Centre for Neuroimaging, London, UK) was used for fMRI data processing. The following steps were included: (1) slicing timing with the 1st slice as a reference; (2) head motion correction; (3) normalization to a Montreal Neurological Institute template at a voxel size of 3 × 3 × 3 mm; (4) smoothing with a 6-mm full-width at half maximum Gaussian kernel. Participants who exhibited head motion exceeding 3 mm of translation or 3° of rotation in any direction were excluded.

#### Statistical analysis

A general linear model was used to model subject specific responses. All six movement parameters (translation; x, y, z; rotation: pitch, roll, yaw) were included in the statistical model. In the first-level (within-group) analyses, “pain > non-pain” contrast was determined, enabling identification of brain regions that were activated or deactivated when participants try to inhibit responses to the pain depicted in the picture. The resulting contrast images were submitted to second-level (between-subject) analysis. One-sample *t*-tests were used to reveal brain activation within each group, and two-sample *t*-tests were performed to detect group differences in brain activation. Finally, both the within-group and between-subject analysis were corrected for multiple comparisons using the false discovery rate (FDR) correction at *p* < 0.05, *k* > 10.

Correlation analyses were conducted to detect correlations between behavioral performance and activation in regions showing significant between-group differences. For the present study, the ROIs of bilateral TPJ were defined as a sphere with a 6-mm radius centered at *x*/*y*/*z* = 39/−60/42 and *x*/*y*/*z* = −33/−60/39 (MNI coordinates) according to the results of our between-group analysis. We used the Marsbar (Matthew Brett et al., 2002) to calculate the contrast values of signal intensity in association with the painful and non-painful stimulation images, which reflect the activation intensity of the ROI. Correlations between TPJ activation and behavioral data (total and subscores of IRI, subscores of CP and subscores of CU traits) were performed using SPSS 18.0 in CD and HC group, respectively.

## Results

### Demographic and behavioral data

The demographics and psychiatric characteristics for the CD group and HC group are reported in Table 1. Age and IQ did not differ significantly between the two groups (both *p* > 0.05). Relative to the HC group, the CD group had a significantly higher AQCV total score (i.e., aggression index) (*t* = 5.67, *p* < 0.001) and SDQ CP trait score (*t* = 3.93, *p* < 0.001) and also had a significantly lower IRI (empathy index) total score (*t* = −2.93, *p* = 0.005) and IRI empathetic concern (*t* = −3.296, *p* = 0.002) and perspective taking subscores (*t* = −2.95, *p* = 0.005). The APSD scores revealed no significant different in CU traits between the two groups. Besides, the two groups also did not differ in terms of reaction time (*t* = 1.05, *p* > 0.05). Pain intensity rating scores were higher for painful (needle penetration with painful expressions) than non-painful (Q-tip touch) stimuli [6.36 ± 2.14 vs. 0.54 ± 0.86, *t*_(130)_ = 20.49, *p* < 0.001], and there were no significant differences between the CD group and HC group in pain intensity rating scores.

**Table 1:** **Demographic and behavioral characteristics of the study cohort**.

**Characteristic**	**CD adolescents (*N* = 30)**	**HCs (*N* = 36)**	***p***
Age (years)	15.07 (0.52)	15.28 (0.45)	0.08
IQ	105.71 (3.51)	107.17 (3.22)	0.08
Painful condition (painful faces)	6.28 (2.09)	6.42 (2.21)	0.78
Painful condition (neutral faces)	3.71 (2.30)	4.04 (2.18)	0.55
Non-painful condition	0.49 (0.68)	0.59 (1.00)	0.63
RTs (ms)	1961.21 (709.14)	1899.86 (599.88)	0.29
IRI total	21.20 (3.09)	23.13 (2.24)	0.005
IRI-Empathic concern	21.31 (3.52)	24.28 (3.74)	0.002
IRI-Perspective-taking	19.98 (4.50)	22.92 (3.60)	0.005
IRI-Personal distress	21.18 (3.31)	21.75 (5.15)	0.59
IRI-Fantasy	22.34 (5.04)	23.59 (4.30)	0.28
AQCV total	90.30 (20.21)	64.39 (16.89)	<0.001
SDQ-conduct problems	4.02 (1.96)	2.36 (1.46)	<0.001
APSD-callous unemotional	5.93 (2.18)	5.39 (1.79)	0.27

*CD, conduct disorder; HCs, healthy controls; RT, reaction time; IRI, Interpersonal Reactivity Index Scale; AQCV, Buss-Perry Aggression Questionnaire; SDQ, Strengths and Difficulties questionnaire; APSD, Antisocial Process Screening Device*.

### Imaging results

#### Group comparison across conditions

**Figure 1:**
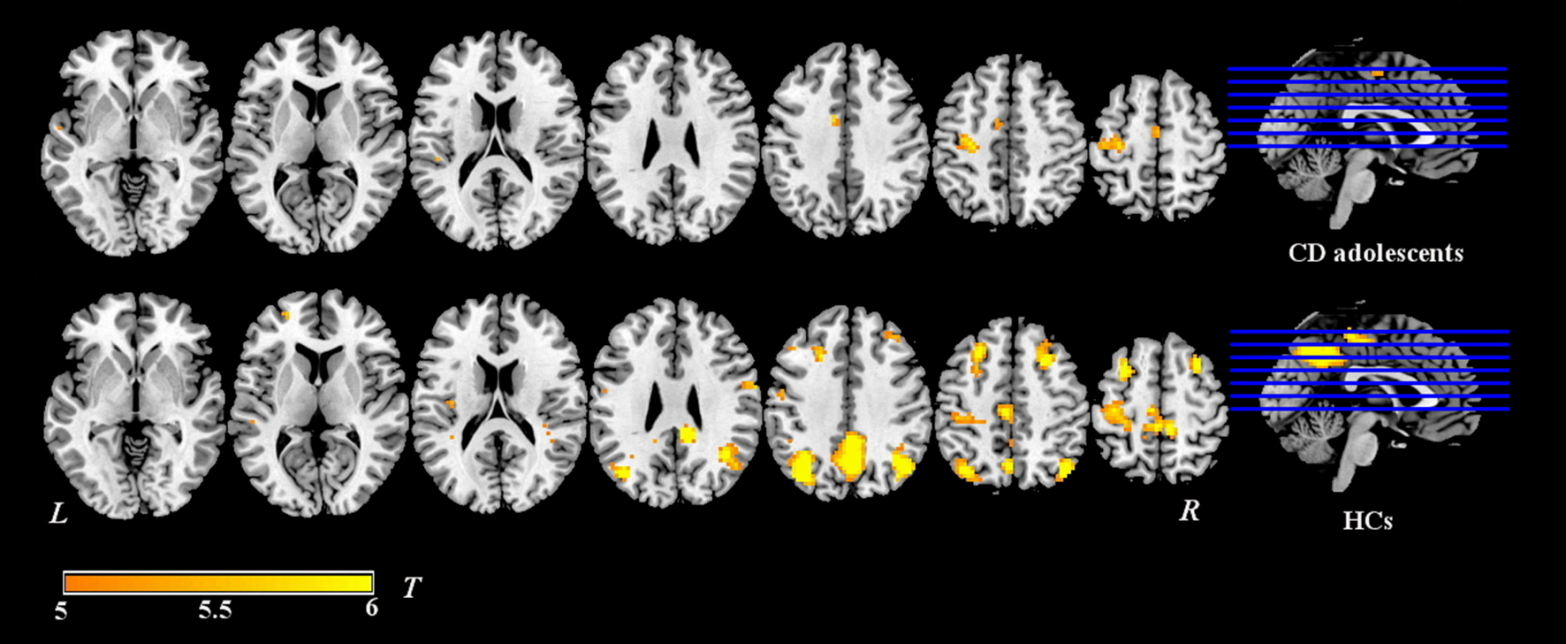
**Activation maps of painful image vs. non-painful image condition in adolescents with CD (***N*** = 30) and HCs (***N*** = 36)**. Relative to the non-painful image condition, in the HCs, the post/midcingulate cortex, insula, precuneus, inferior parietal lobule, supramarginal gyrus, superior frontal gyrus, middle frontal gyrus, precentral gyrus, fusiform gyrus, middle temporal gyrus, and superior temporal gyrus were significantly activated in the painful image condition. In the CD group, the midcingulate gyrus, insula, precentral gyrus, and superior temporal gyrus were significantly activated in the painful image stimulation.Significance was determined by one-sample *t*-tests; significance level was set to *p* < 0.05 (false discovery rate corrected). Color bar signifies *t*-values. L, left; R, right; CD, conduct disorder, HCs, healthy controls.

Group-level whole-brain analysis of condition (painful vs. non-painful image) main-effect results are reported in Table 2 and Figure 1. Relative to the non-painful image condition, in the HCs, the right post/midcingulate cortex, right insula, right precuneus, bilateral inferior parietal lobule, right supramarginal gyrus, right superior frontal gyrus, bilateral middle frontal gyrus, right precentral gyrus, right fusiform gyrus, right middle temporal gyrus, and right superior temporal gyrus were significantly activated in the painful image condition (FDR correction, *p* < 0.05, *k* > 10 vs. non-pain images). In the CD group, the left midcingulate gyrus, left insula, left precentral gyrus, and left superior temporal gyrus were significantly activated in the painful image stimulation (FDR correction, *p* < 0.05, *k* > 10 vs. non-pain images).

**Table 2:** **Activations differing between painful and non-painful images**.

**Location**	**Side**	**BA**	**MNI coordinates**			**Cluster size**	***t*****-value**
			***X***	***Y***	***Z***		
**HC GROUP**							
Post/midcingulate cortex	R	31	12	−39	30	145	8.68
Insula	R	13	36	−33	18	45	5.07
Precuneus	R	7	6	−60	42	517	8.30
Inferior parietal lobule	R		42	−63	39	314	8.34
	L		−33	−60	39	324	6.00
Supramarginal gyrus	R		39	−54	27	46	6.77
Superior frontal gyrus	R		30	15	54	185	7.01
Middle frontal gyrus	R	8	27	30	51	389	5.6
	L		−24	54	9	74	5.62
Precentral gyrus	R		60	−3	30	100	6.00
Fusiform gyrus	R	20	54	−30	−24	112	5.21
Middle temporal gyrus	R		60	−45	−9	59	5.02
Superior temporal gyrus	R	13	42	−45	18	159	5.27
**CD GROUP**							
Midcingulate gyrus	L	24	−9	0	42	118	6.22
Insula	L		−40	−30	18	39	5.40
Precentral gyrus	L		−27	−21	51	347	7.00
Superior temporal gyrus	L		−57	−6	0	27	5.13

*BA, Brodmann area; MNI, Montreal Neurological Institute; CD, conduct disorder; HCs, healthy controls. All p < 0.05, false discovery rate corrected, k > 10*.

#### Between-group analysis

**Figure 2:**
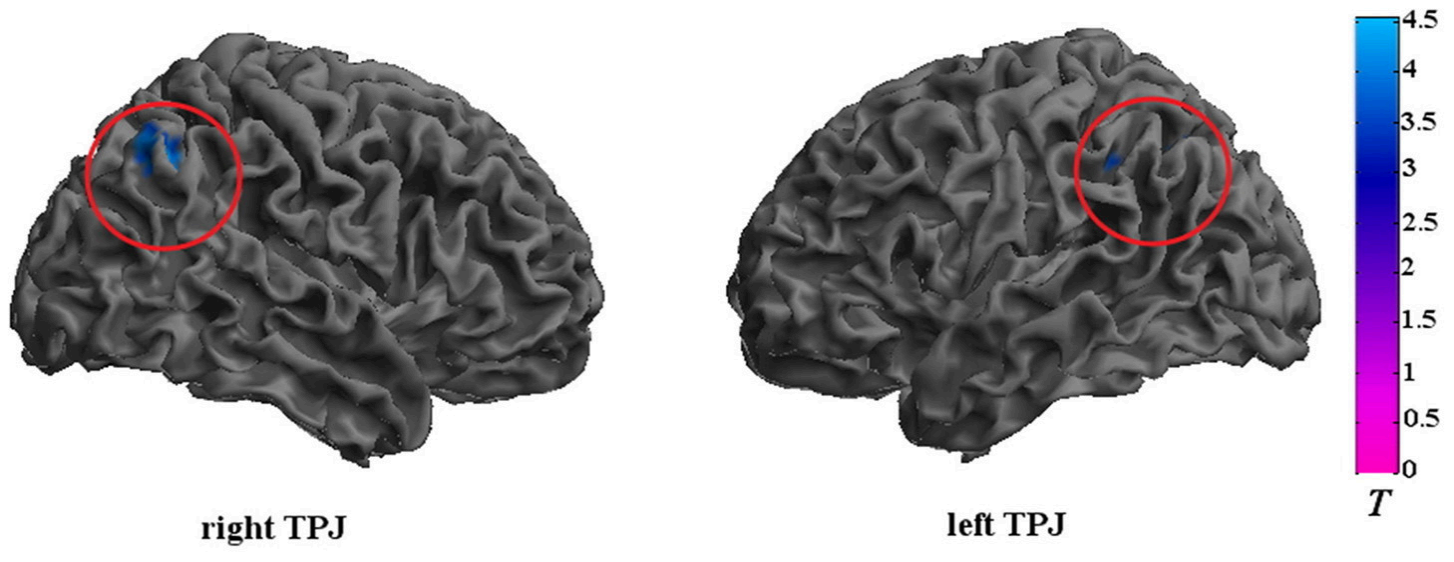
**Brain regions that exhibited reduced activation in adolescents with CD compared to HCs**. The CD group showed significantly reduced activation in the bilateral TPJ. Significance was determined by two-sample *t*-tests; significance level was set to *p* < 0.05 (false discovery rate corrected). Color bar signifies *t*-values. TPJ, temporoparietal junction.

The CD group showed significantly reduced activation in the bilateral TPJ (FDR correction, *p* < 0.05, *k* > 10 vs. HCs) in relative to the HC group. The detailed results of the between-group analysis are reported in Table 3 and Figure 2.

**Table 3:** **Reduced TPJ activation in painful image condition, relative to non-painful image condition, in CD adolescents compared to HCs**.

**TPJ side**	**BA**	**MNI coordinates**			**Cluster size**	***t*****-value**
		***X***	***Y***	***Z***		
R	39/40	39	−60	42	90	4.54
L	39	−33	−60	39	40	4.51

*CD, conduct disorder; HCs, healthy controls; BA, Brodmann area; MNI, Montreal Neurological Institute. All p < 0.05, false discovery rate corrected, k > 10*.

#### Correlation analysis results

No significant correlation results were detected between empathic response magnitude in the bilateral TPJ and subjective ratings of CP, CU traits, or empathy.

## Discussion

To our knowledge, this is the first study to investigate the neural substrate of affective and cognitive empathy in a large sample of adolescents with “pure” CD. Both the regions associated with affective empathy (insula and midcingulate gyrus) and regions associated with cognitive empathy (postcingulate gyrus, precuneus, and TPJ) were activated in the HC group while viewing painful stimulation images, relative to non-painful stimulation images. Relative to HCs, the CD group exhibited dampened hemodynamic empathic responses in the bilateral TPJ, a region associated with cognitive empathy. However, we did not observe group differences in regions associated with affective empathy, including the anterior insula, inferior frontal gyrus, and middle ACC.

Interestingly, we observed reduced hemodynamic responses in the TPJ in adolescents with CD, a finding that has not been reported previously. One study (Sebastian et al., 2012) exploring the affective theory of mind (cognitive empathy) in children with CP suggested that the children with CP showed hypoactivity in amygdala and anterior insula. To our knowledge, this inconsistence might because of the differences of samples, in that study, common comorbidities (ADHD, general anxiety disorder, depression and substance abuse) were not used as exclusion criteria (Decety and Lamm, 2006; Sebastian et al., 2012). In other related studies (Decety et al., 2009; Lockwood et al., 2013; Schwenck et al., 2017), tasks evoking affective empathy were used to explore the neural substrate of empathy, the participants either observed video clips of body parts being harmed passively or performed a distraction task, in a more recently study (Schwenck et al., 2017), a gambling task which was frequently used to assess affective empathy were recruited. Hence, these prior studies may have been mainly exploring bottom-up affective empathy but not top-down cognitive empathy. Previously, cognitive empathy has been associated with several brain regions, including ventral and dorsal aspects of the medial prefrontal cortex, superior temporal sulcus, precuneus, and TPJ (Shamay-Tsoory, 2013; Raz et al., 2014).

The TPJ is a region encompassing the supramarginal gyrus, caudal parts of the superior temporal gyrus, and dorsal-rostral parts of the occipital gyri. It is a heteromodal association cortex, which integrates input from the lateral and posterior thalamus, as well as visual, auditory, somaesthetic, and limbic area (Decety and Lamm, 2007). The TPJ has often been considered to be involved with cognitive empathy (Shamay-Tsoory et al., 2009; Shamay-Tsoory, 2011, 2013; Raz et al., 2014), this is, the making of inferences regarding the mental state of others (Shamay-Tsoory, 2013; Raz et al., 2014). Some studies have suggested that the TPJ may be related to the perception process of socially related cues that communicate the mental states of others (Frith and Frith, 2003; Decety and Lamm, 2006, 2007), while other studies have suggested that the TPJ may be important for making inferences about others' beliefs and distinguishing the self from others (Ruby and Decety, 2004; Singer and Lamm, 2009; Cheng et al., 2010; Shamay-Tsoory, 2011). Altogether, the TPJ seems to be a component of a neural network which allows for the mental separation of one's own perspective from that of others, thus enabling us to detangle our own feelings from those observed from others (Schulte-Rüther et al., 2008). The reduced TPJ activation observed in our CD group might reflect the adolescents with CD having a deficiency in inferring others' mental states, which would fit well previously reported CD-related behavioral findings (Cohen and Strayer, 1996; Blair et al., 2001), as well as with the present study behavioral data. However, distinct empathic neural responses in TPJ between two groups didn't necessarily result in different conscious subjective ratings of others' pain intensity, as indicated by measures of subjective ratings in current work. This might reflect that the empathy-related TPJ activity observed here was unconscious responses.

Significant distinct hemodynamic responses have been observed in children with CP during pain-related processing in regions associated with affective empathy in previous studies, including the inferior frontal gyrus, anterior insula, and the middle ACC. Lockwood et al. (2013) found reduced activation of the ACC, anterior insula, and inferior frontal gyrus in children with CP. Decety et al. (2009) found increased empathy-associated responses in anterior midcingulate cortex, right middle cingulate cortex, and middle insula in adolescents with aggressive CD (*p* < 0.005, uncorrected). Sebastian et al. (2012) found the children with CP showed reduced empathic responses in anterior insula and amygdala, and Schwenck et al. (2017) found that the brain regions associated with affective empathy weren't activated in boys with CP in a gambling task. However, in the present study, the adolescents with “pure” CD did not exhibit activation different from that observed in HCs in brain regions associated with affective empathy. Their relatively low CU trait scores might account for this negative finding in our study.

Indeed, we did not find any significant differences in CU traits between the CD group and HCs. When Cheng et al. (2012) compared electrophysiological responses between CD youth with high vs. low CU traits, they found that only the central late positive potential, which reflects a late cognitive evaluation component (Fan and Han, 2008; Decety and Michalska, 2010), was reduced in the low-CU group, whereas both the late positive potential and the frontal N120 component, which is associated with early affective arousal (Fan and Han, 2008; Decety and Michalska, 2010), were reduced in the high-CU group. The findings of Cheng et al.'s study suggest that individuals with low CU traits might have a selective cognitive empathy deficiency. Our results support this inference to some extent. Although the CU trait scores of the CD adolescents in our study were similar to those of HCs, their scores were quite variable indicating that our CD group was heterogeneous with respect to CU traits. The neural substrate in CD adolescents with low CU traits needs to be investigated further. Several studies have illustrated us that CU traits do not necessarily accompany CD (Blair et al., 2006; Frick and Dickens, 2006). Rather, CU traits *per se* have been related to empathic responses in brain regions associated with affective empathy (Lockwood et al., 2013; Michalska et al., 2015). Hence, CU traits should perhaps be taken into consideration in future studies of CD.

There are several potential limitations of this work that should be mentioned. Firstly, the CU trait scores of our CD group were not treated as a variable due to the limited sample size. Secondly, our behavioral paradigm cannot be used to clarify CD-associated differences related to different components of empathy, such as emotional cognition, emotional perspective taking, and affective responsiveness (Decety and Jackson, 2004). A more detailed paradigm will be necessary to distinguish among the different aspects of empathy. Finally, the present cohort consisted only of males. Sex differences in the neural correlates of CD have been reported (Michalska et al., 2015). Therefore, the present results may not generalize to females.

In conclusion, the present research provides insights into the neural substrate of affective and cognitive empathy in adolescents with “pure” CD. Both brain regions associated with cognitive empathy and brain regions associated with affective empathy were activated in the HC group with a pain-judgment empathy task. The “pure” CD adolescents had dampened hemodynamic empathic responses in the bilateral TPJ, a region associated with cognitive empathy, suggesting that adolescents with “pure” CD might have a deficiency in cognitive empathy. Besides, atypical affective empathic responses were not observed in the CD group, which might result from the relatively low CU traits of our sample.

## Author contributions

SY designed the study; YJ, YG, and QW performed the study; QM, XW, and WY helped to draft the manuscript; DD analyzed the data and wrote the manuscript.

## Funding

This work was supported by grants from the Natural Science Foundation of China (81471384 to SY); the National Key Technologies R&D Program in China's 11th 5-year plan (2009BAI77B02 to SY); and the Specialized Research Fund for the Doctoral Program of Higher Education (20130162110043 to SY), as well as the construct program for key disciplines in Hunan province.

### Conflict of interest statement

The authors declare that the research was conducted in the absence of any commercial or financial relationships that could be construed as a potential conflict of interest.

## References

[B1] APA (2000). Diagnostic and Statistical Manual of Mental Disorders DSM-IV-TR, 4th Edn. Washington, DC: American Psychiatric Publishing.

[B2] Bernhardt B. C. Singer T. (2012). The neural basis of empathy. Neuroscience 35:1. 10.1146/annurev-neuro-062111-150536 22715878

[B3] Blair R. J. Colledge E. Mitchell D. (2001). Somatic markers and response reversal: is there orbitofrontal cortex dysfunction in boys with psychopathic tendencies? J. Abnorm. Child Psychol. 29, 499–511. 10.1023/A:1012277125119 11761284

[B4] Blair R. J. Peschardt K. S. Budhani S. Mitchell D. G. Pine D. S. (2006). The development of psychopathy. J. Child Psychol. Psychiatry 47, 262–276. 10.1111/j.1469-7610.2006.01596.x 16492259

[B5] Blair R. J. R. (2005). Responding to the emotions of others: dissociating forms of empathy through the study of typical and psychiatric populations. Conscious. Cogn. 14, 698–718. 10.1016/j.concog.2005.06.004 16157488

[B6] Buss A. H. Perry M. (1992). The aggression questionnaire. J. Pers. Soc. Psychol. 63:452. 10.1037/0022-3514.63.3.452 1403624

[B7] Cheng Y. Chen C. Lin C. P. Chou K. H. Decety J. (2010). Love hurts: an fMRI study. Neuroimage 51, 923–929. 10.1016/j.neuroimage.2010.02.047 20188182

[B8] Cheng Y. Hung A. Y. Decety J. (2012). Dissociation between affective sharing and emotion understanding in juvenile psychopaths. Dev. Psychopathol. 24, 623–636. 10.1017/S095457941200020X 22559135

[B9] Christov-Moore L. Simpson E. A. Coudé G. Grigaityte K. Iacoboni M. Ferrari P. F. (2014). Empathy: gender effects in brain and behavior. Neurosci. Biobehav. Rev. 46(Pt 4), 604–627. 10.1016/j.neubiorev.2014.09.001 25236781 PMC5110041

[B10] Cohen D. Strayer J. (1996). Empathy in conduct-disordered and comparison youth. Dev. Psychol. 32:988 10.1037/0012-1649.32.6.988

[B11] Davis M. H. (1980). A multidimensional approach to individual differences in empathy. JSAS Catalog Sel. Doc. Psychol. 10:85.

[B12] Davis M. H. (1983). Measuring individual differences in empathy: evidence for a multidimensional approach. J. Pers. Soc. Psychol. 44:113 10.1037/0022-3514.44.1.113

[B13] Decety J. Jackson P. L. (2004). The functional architecture of human empathy. Behav. Cogn. Neurosci. Rev. 3, 71–100. 10.1177/1534582304267187 15537986

[B14] Decety J. Lamm C. (2006). Human empathy through the lens of social neuroscience. ScientificWorldJournal 6, 1146–1163. 10.1100/tsw.2006.221 16998603 PMC5917291

[B15] Decety J. Lamm C. (2007). The role of the right temporoparietal junction in social interaction: how low-level computational processes contribute to meta-cognition. Neuroscientist 13, 580–593. 10.1177/1073858407304654 17911216

[B16] Decety J. Michalska K. J. (2010). Neurodevelopmental changes in the circuits underlying empathy and sympathy from childhood to adulthood. Dev. Sci. 13, 886–899. 10.1111/j.1467-7687.2009.00940.x 20977559

[B17] Decety J. Michalska K. J. Akitsuki Y. Lahey B. B. (2009). Atypical empathic responses in adolescents with aggressive conduct disorder: a functional MRI investigation. Biol. Psychol. 80, 203–211. 10.1016/j.biopsycho.2008.09.004 18940230 PMC2819310

[B18] Derntl B. Finkelmeyer A. Eickhoff S. Kellermann T. Falkenberg D. I. Schneider F.. (2010). Multidimensional assessment of empathic abilities: neural correlates and gender differences. Psychoneuroendocrinology 35, 67–82. 10.1016/j.psyneuen.2009.10.006 19914001

[B19] Eisenberg N. Cumberland A. Guthrie I. K. Murphy B. C. Shepard S. A. (2005). Age changes in prosocial responding and moral reasoning in adolescence and early adulthood. J. Res. Adolesc. 15, 235–260. 10.1111/j.1532-7795.2005.00095.x 20592955 PMC2893741

[B20] Fan Y. Duncan N. W. de Greck M. Northoff G. (2011). Is there a core neural network in empathy? An fMRI based quantitative meta-analysis. Neurosci. Biobehav. Rev. 35, 903–911. 10.1016/j.neubiorev.2010.10.009 20974173

[B21] Fan Y. Han S. (2008). Temporal dynamic of neural mechanisms involved in empathy for pain: an event-related brain potential study. Neuropsychologia 46, 160–173. 10.1016/j.neuropsychologia.2007.07.023 17825852

[B22] First M. B. Spitzer R. L. Gibbon M. Williams J. B. (2001). Structured Clinical Interview for DSM-IV-TR Axis I Disorders—Non-Patient Edition. New York, NY: New York State Psychiatric Institute.

[B23] Frick P. Hare R. (2001). Antisocial Process Screening Device. Toronto: Multi-Health Systems.

[B24] Frick P. J. Dickens C. (2006). Current perspectives on conduct disorder. Curr. Psychiatry Rep. 8, 59–72. 10.1007/s11920-006-0082-3 16513044

[B25] Frith U. Frith C. D. (2003). Development and neurophysiology of mentalizing. Philos. Trans. R. Soc. Lond. B Biol. Sci. 358, 459–473. 10.1098/rstb.2002.1218 12689373 PMC1693139

[B26] Gong Y. Cai T. (1993). Wechsler Intelligence Scale for Children, Chinese Revision (C-WISC). Changsha: Map Press Hunan.

[B27] Han S. Fan Y. Mao L. (2008). Gender difference in empathy for pain: an electrophysiological investigation. Brain Res. 1196, 85–93. 10.1016/j.brainres.2007.12.062 18221733

[B28] Han S. Fan Y. Xu X. Qin J. Wu B. Wang X.. (2009). Empathic neural responses to others' pain are modulated by emotional contexts. Hum. Brain Mapp. 30, 3227–3237. 10.1002/hbm.20742 19235883 PMC6870998

[B29] Jiang Y. Guo X. Zhang J. Gao J. Wang X. Situ W.. (2015). Abnormalities of cortical structures in adolescent-onset conduct disorder. Psychol. Med. 45, 3467–3479. 10.1017/S0033291715001361 26189512

[B30] Lamm C. Decety J. Singer T. (2011). Meta-analytic evidence for common and distinct neural networks associated with directly experienced pain and empathy for pain. Neuroimage 54, 2492–2502. 10.1016/j.neuroimage.2010.10.014 20946964

[B31] Lockwood P. L. Sebastian C. L. McCrory E. J. Hyde Z. H. Gu X. De Brito S. A.. (2013). Association of callous traits with reduced neural response to others' pain in children with conduct problems. Curr. Biol. 23, 901–905. 10.1016/j.cub.2013.04.018 23643836 PMC3918856

[B32] Loeber R. Burke J. D. Lahey B. B. Winters A. Zera M. (2000). Oppositional defiant and conduct disorder: a review of the past 10 years, part I. J. Am. Acad. Child Adolesc. Psychiatry 39, 1468–1484. 10.1097/00004583-200012000-00007 11128323

[B33] Matthew Brett J.-L. A. Valabregue R. Poline J.-B. (2002). Region of interest analysis using an SPM toolbox [*abstract*], in Presented at the 8th International Conference on Functional Mapping of the Human Brain (Sendai). Available on CD-ROM in NeuroImage Vol 16, No 2.

[B34] Michalska K. J. Zeffiro T. A. Decety J. (2015). Brain response to viewing others being harmed in children with conduct disorder symptoms. J. Child Psychol. Psychiatry. 57, 510–519. 10.1111/jcpp.12474 26472591 PMC4789171

[B35] Oldfield R. C. (1971). The assessment and analysis of handedness: the Edinburgh inventory. Neuropsychologia 9, 97–113. 10.1016/0028-3932(71)90067-4 5146491

[B36] Olsson M. (2009). DSM diagnosis of conduct disorder (CD)—a review. Nord. J. Psychiatry 63, 102–112. 10.1080/08039480802626939 19085560

[B37] Preston S. D. De Waal F. B. (2002). Empathy: its ultimate and proximate bases. Behav. Brain Sci. 25, 1–20. 10.1017/S0140525X02000018 12625087

[B38] Raz G. Jacob Y. Gonen T. Winetraub Y. Flash T. Soreq E.. (2014). Cry for her or cry with her: context-dependent dissociation of two modes of cinematic empathy reflected in network cohesion dynamics. Soc. Cogn. Affect. Neurosci. 9, 30–38. 10.1093/scan/nst052 23615766 PMC3871736

[B39] Rizzolatti G. (2005). The mirror neuron system and its function in humans. Anat. Embryol. 210, 419–421. 10.1007/s00429-005-0039-z 16222545

[B40] Rubia K. (2011). “Cool” inferior frontostriatal dysfunction in attention-deficit/hyperactivity disorder versus “hot” ventromedial orbitofrontal-limbic dysfunction in conduct disorder: a review. Biol. Psychiatry 69, e69–e87. 10.1016/j.biopsych.2010.09.023 21094938

[B41] Ruby P. Decety J. (2004). How would you feel versus how do you think she would feel? A neuroimaging study of perspective-taking with social emotions. J. Cogn. Neurosci. 16, 988–999. 10.1162/0898929041502661 15298786

[B42] Rueckert L. Naybar N. (2008). Gender differences in empathy: the role of the right hemisphere. Brain Cogn. 67, 162–167. 10.1016/j.bandc.2008.01.002 18295950

[B43] Schulte-Rüther M. Markowitsch H. J. Shah N. J. Fink G. R. Piefke M. (2008). Gender differences in brain networks supporting empathy. Neuroimage 42, 393–403. 10.1016/j.neuroimage.2008.04.180 18514546

[B44] Schwenck C. Ciaramidaro A. Selivanova M. Tournay J. Freitag C. M. Siniatchkin M. (2017). Neural correlates of affective empathy and reinforcement learning in boys with conduct problems: fMRI evidence from a gambling task. Behav. Brain Res. 320, 75–84. 10.1016/j.bbr.2016.11.037 27888020

[B45] Schwenck C. Mergenthaler J. Keller K. Zech J. Salehi S. Taurines R.. (2012). Empathy in children with autism and conduct disorder: Group-specific profiles and developmental aspects. J. Child Psychol. Psychiatry 53, 651–659. 10.1111/j.1469-7610.2011.02499.x 22118246

[B46] Sebastian C. L. McCrory E. J. Cecil C. A. Lockwood P. L. De Brito S. A. Fontaine N. M.. (2012). Neural responses to affective and cognitive theory of mind in children with conduct problems and varying levels of callous-unemotional traits. Arch. Gen. Psychiatry 69, 814–822. 10.1001/archgenpsychiatry.2011.2070 22868935

[B47] Shamay-Tsoory S. G. (2011). The neural bases for empathy. Neuroscientist 17, 18–24. 10.1177/1073858410379268 21071616

[B48] Shamay-Tsoory S. G. (2013). Dynamic functional integration of distinct neural empathy systems. Soc. Cogn. Affect. Neurosci. 9, 1–2. 10.1093/scan/nst107 23956080 PMC3871737

[B49] Shamay-Tsoory S. G. Aharon-Peretz J. Perry D. (2009). Two systems for empathy: a double dissociation between emotional and cognitive empathy in inferior frontal gyrus versus ventromedial prefrontal lesions. Brain 132(Pt 3), 617–627. 10.1093/brain/awn279 18971202

[B50] Singer T. Lamm C. (2009). The social neuroscience of empathy. Ann. N. Y. Acad. Sci. 1156, 81–96. 10.1111/j.1749-6632.2009.04418.x 19338504

[B51] Whitmore E. A. Mikulich S. K. Thompson L. L. Riggs P. D. Aarons G. A. Crowley T. J. (1997). Influences on adolescent substance dependence: conduct disorder, depression, attention deficit hyperactivity disorder, and gender. Drug Alcohol. Depend. 47, 87–97. 10.1016/S0376-8716(97)00074-4 9298330

[B52] Wied M. Goudena P. P. Matthys W. (2005). Empathy in boys with disruptive behavior disorders. Journal of Child Psychology and Psychiatry 46, 867–880. 10.1111/j.1469-7610.2004.00389.x 16033635

[B53] Xu X. Zuo X. Wang X. Han S. (2009). Do you feel my pain? Racial group membership modulates empathic neural responses. J. Neurosci. 29, 8525–8529. 10.1523/JNEUROSCI.2418-09.2009 19571143 PMC6665679

[B54] Yao S. Zhang C. Zhu X. Jing X. McWhinnie C. M. Abela J. R. (2009). Measuring adolescent psychopathology: psychometric properties of the self-report strengths and difficulties questionnaire in a sample of Chinese adolescents. J. Adolesc. Health 45, 55–62. 10.1016/j.jadohealth.2008.11.006 19541250

[B55] Zhang J. Li B. Gao J. Shi H. Wang X. Jiang Y.. (2015). Impaired frontal-basal ganglia connectivity in male adolescents with conduct disorder. PLoS ONE 10:e0145011. 10.1371/journal.pone.0145011 26658732 PMC4682835

